# “Everything is kind of the same except my mind is with me”: exploring cannabis substitution in a sample of adults in early recovery from an opioid or stimulant addiction

**DOI:** 10.1186/s12954-024-01002-0

**Published:** 2024-04-20

**Authors:** Corinne A. Beaugard, Alexander Y. Walley, Maryann Amodeo

**Affiliations:** 1 Boston University School of Social Work, 264 Bay State Road, Boston, MA 02215 USA; 2 Boston Medical Center, Grayken Center for Addiction, Clinical Addiction Research and Education Unit, Section of General Internal Medicine, Department of Medicine, Boston University Chobanian & Avedisian School of Medicine, 801 Massachusetts Ave, 2nd Floor, Boston, MA 02118 USA; 3 Department of Psychiatry, Boston Medical Center Crosstown Center, 4th Floor 801 Massachusetts Avenue, 02118 Boston, MA USA

## Abstract

**Background:**

Recovery from addiction is frequently equated with abstinence. However, some individuals who resolve an addiction continue to use substances, including via substitution (i.e., increased use of one substance after eliminating/ reducing another). Substitution may play a distinct role during early recovery (≤ 1 year), as this period is marked by dramatic change and adjustment. Cannabis is one of the most used substances and is legal for medical and recreational use in an increasing number of states. Consequently, cannabis an increasingly accessible substitute for substances, like fentanyl, heroin, cocaine and methamphetamine, with higher risk profiles (e.g., associated with risk for withdrawal, overdose, and incarceration).

**Methods:**

Fourteen participants reported that they had resolved a primary opioid or stimulant addiction and subsequently increased their cannabis use within the previous 12 months. Using grounded theory, the interviewer explored their experiences of cannabis use during early recovery. Data were analyzed in three stages: line by line coding for all text related to cannabis use and recovery, focused coding, and axial coding to generate a theory about recovery with cannabis substitution. The motivational model of substance use provided sensitizing concepts.

**Results & discussion:**

The final sample included eight men and six women ranging in age from 20 to 50 years old. Three participants resolved an addiction to methamphetamine and the remaining 11, an addiction to opioids. Participants explained that cannabis was appealing because of its less harmful profile (e.g., no overdose risk, safe supply, few side effects). Participants’ primary motives for cannabis use included mitigation of psychiatric symptoms, withdrawal/ cravings, and boredom. While cannabis was effective toward these ends, participants also reported some negative side effects (e.g., decreased productivity, social anxiety). All participants described typical benefits of recovery (e.g., improved self-concept, better relationships) while continuing to use cannabis. Their experiences with and beliefs about substitution suggest it can be an effective strategy for some individuals during early recovery.

**Conclusions:**

Cannabis use may benefit some adults who are reducing their opioid or stimulant use, especially during early recovery. The addiction field’s focus on abstinence has limited our knowledge about non-abstinent recovery. Longitudinal studies are needed to understand the nature of substitution and its impact on recovery over time.

## Background

Most addiction treatment settings, mutual aid groups, and research on recovery posit that recovery is built upon a foundation of abstinence from psychoactive drugs, excluding nicotine and prescription medication [1, 2]. This operationalization of recovery aligns with the Substance Abuse and Mental Health Services Administration (SAMHSA)’ definition which states, “[Recovery is] a process of change through which individuals improve their health and wellness, live a self-directed life, and strive to reach their full potential” [3]. And while this definition suggests that recovery incorporates holistic growth, the SAMHSA text later specifies that “abstinence from the use of alcohol, illicit drugs, and non-prescribed medications is the goal for those with addictions” [3]. This standard orientation toward recovery excludes individuals who resolved their addictions without abstinence, thus limiting the field’s capacity to understand and support this potentially large and heterogenous population. Among individuals who are in non-abstinence recovery, a subset “substitute,” or increase use of one substance following the decreased use or cessation of another. Motives for substitution vary and include the relative availability or cost, side effects, and risks of the original and substitute substances [4–10].

Second to alcohol and tobacco, cannabis is the most frequently used substance [3]. Cannabis is perceived to be less harmful than other substances, and consequent to its increasing legalization for medical and recreational purposes, has been viewed more favorably by the public [11–13]. Research on cannabis substitution suggests it can be an effective strategy to decrease more harmful substance use (e.g., crack cocaine, opioids, alcohol, or prescription drugs) in part because it has less adverse side effects and less withdrawal potential than other drugs [7, 9, 14]. Paradoxically, in clinical samples, cannabis use clustered with more active and severe use of other substances. For example, in one study cannabis substitution was associated with a 27% reduction in odds of abstinence from other drugs or alcohol [15]. In another, cannabis use was three times higher amongst those who returned to cocaine use; however, cannabis use was not associated with a return to heroin use [16].

There is little research on cannabis substitution amongst individuals in recovery– likely due to the addiction field’s normative conflation of abstinence and recovery [1, 17–20]. Substitution during recovery, or after resolving an addiction, may function similarly to substitution during an addiction; however, there is no research that examines the experience and function of substitution during recovery. Early recovery, often defined as one year [19, 21], is a unique period marked by dramatic change in behavior and lifestyle, and experiences during this period are associated with future recovery outcomes [22–24]. Because early recovery is distinct in the magnitude of change that occurs across many domains (e.g., professional, family, community, physical and mental health), substitution might be more common or have specific functions during this period [19].

This study was designed to address the gap in research on substitution among people in recovery. Exploring how people in early recovery from an opioid or stimulant addiction experience cannabis substitution can provide insight on whether increased use of one substance supports recovery from another. The primary aims of this study were: (1) to identify individuals’ motives for cannabis use after resolving an opioid or stimulant addiction (2), to describe individuals’ experiences using cannabis, and (3) to understand whether cannabis substitution and addiction resolution are compatible.

## Methods

### Participants and recruitment

Data for this study were collected from a community sample of people who resolved a stimulant or opioid addiction in the previous 12 months and subsequently increased their cannabis use. Additional eligibility requirements included being at least 18 years old, English language fluency, US residence, and the ability to consent.

**Fig. 1:**
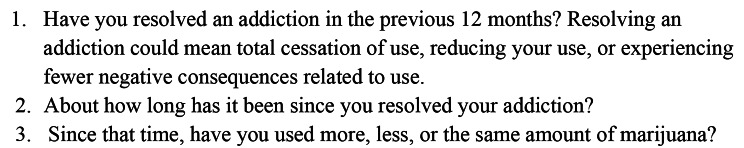
Interview Screening Questions

For the purposes of recruitment and clarity of construct, “resolved an addiction” was chosen instead of “recovery” so that potential participants did not exclude themselves based on an association between recovery and abstinence. The term “addiction” was used rather than “substance use disorder” so that people could identify with this more common phrase rather than a formal diagnostic term. The authors posted recruitment materials on Facebook and Reddit pages related to addiction and recovery. The materials opened with, “Are you in the first 12 months of resolving an opioid or stimulant addiction?” and stated that the study was designed to, “understand more about non-abstinence recovery for people who resolved an opioid or stimulant addiction and currently use cannabis.” Most participants described themselves as in “recovery,” which is how they will be described in the results. During the phone screening, participants stated which addiction they resolved, their current substance use, and whether their cannabis use increased, decreased, or stayed the same after resolving their addiction (see Fig. 1).

The first author interviewed 14 participants over Zoom. Participants resided across the US, in the Northeast, Southeast, Midwest, and Pacific Northwest regions. The authors did not collect any identifying information about participants and chose participant pseudonyms that reflected each participant’s self-reported racial and ethnic identities. Before the interview, the first author reviewed the consent document with each participant and received verbal consent. Interviews lasted approximately 1 h, and participants received a $30 Amazon electronic gift card upon completion. The Boston University Charles River Campus IRB approved this study.

### Interviews

The interview guide included questions about participants’ substance use routines, experiences that prompted cannabis use, and the effect of cannabis on their recovery. The interview opened with the question, “Since you’ve been in recovery, what substances have you used?” In many cases, participants described their substance use and provided context for this use. Building on their context, probes included questions such as, “What types of things make you want to use cannabis?” or, in response to a specific example of cannabis use, “Can you describe what was going on before you used cannabis?” After participants thoroughly described motives for cannabis use, follow up prompts aimed to understand their experience using cannabis, for example, “How did you feel after you used cannabis?” Of note, prompts reflected the participants’ language about cannabis and their mechanisms of use, such that the phrasing was modified for each participant (e.g., “Can you describe what was going on before you smoked pot?”).

### Constructivist grounded theory and the motivational model of substance use

The intent of constructivist grounded theory is to create new theory with the acknowledgment that research is inevitably influenced by researchers’ knowledge about the world and pre-existing theories. Thus, theories can be integrated into this methodology for the purpose of “sensitizing concepts,” which inform the research, rather than direct it. Sensitizing concepts help the researcher find “a place to *start* inquiry, not to *end* it” (p. 31) [25].

The motivational model of substance use is a framework that proposes reasons that people use substances and includes four primary motives: to cope with psychological discomfort (e.g., affect regulation), to be comfortable in social situations, to experience enhancement (e.g., to increase pleasure), and to conform (e.g., to align with peer expectations) [26]. Coping and enhancement motives are generally associated with more frequent substance use, as well as more severe substance-related problems [27–29]. During analysis, this theoretical model was used to suggest sensitizing concepts related to substitution motives.

### Data analysis: grounded theory

Interviews were recorded and transcribed. In traditional grounded theory research, interviews are conducted and analyzed simultaneously [25]. This study took a modified grounded theory approach. The first author conducted and analyzed three interviews simultaneously and drafted an initial codebook from these interviews; they analyzed the remaining 11 interviews together. A second coder [MA] independently coded 11 transcripts using the codebook. The two coders discussed discrepancies until consensus was reached.

Following a grounded theory approach, authors coded the interviews in three stages [25]. The first stage involved line-by-line coding for all text related to participants’ substance use after resolving a primary addiction, experiences using cannabis, and beliefs about the effects of cannabis on their recovery. During initial coding, the motivational model provided sensitizing concepts (i.e., the four motives for substance use) [30]. During focused coding, the authors identified the salient processes and actions that explained motives for cannabis use, the physical and psychological effects of cannabis, and the role of cannabis use in participants’ lives. Finally, during axial coding, authors identified the relationships across themes to build an explanatory model for cannabis substitution.

### Methodological integrity

The interviewer [CAB] had worked in addiction settings, was trained in qualitative methods, and had conducted previous interviews with people in recovery, as well as people with current addictions. During this study, a qualitative scholar provided methodological supervision related to study design, interviewing, and data analysis. Co-authors [AYW and MA], both experts in addiction treatment, offered guidance on the inclusion criteria, recruitment strategies, the interview protocol, and analysis.

Authors engaged with reflexivity by writing memos after each interview and meeting to discuss the interviews and coding to mitigate bias during analysis. Writing after each interview allowed authors to disentangle participants’ construction of the concepts from their impressions and anticipated responses [31]. As the interviews and analyses progressed, it became apparent that the experience of non-abstinence recovery with cannabis substitution was different from what had been expected. This realization affirmed the importance of this methodology; a different approach (e.g., surveys or more structured interviews), would have limited participants’ ability to shape the preliminary theory of non-abstinence recovery with substitution.

## Results

Participants described their experiences of increasing cannabis use after resolving a primary opioid or stimulant addiction (See Table 1). Most participants were non-Hispanic White [11], two participants were Hispanic, and one participant was Black Somali. The sample included eight men and six women ranging in age from 20 to 50 years old. Three participants resolved an addiction to methamphetamine and the remaining 11, an addiction to opioids (primarily fentanyl, reflecting the current drug supply). None of the participants reported their cannabis use was exclusively for medical purposes and only one participant reported access to medical cannabis. The major themes and processes that emerged from interviews included: (1) cannabis is a better alternative: relatively safe, legally accessible, & socially acceptable; (2) cannabis use is motive driven; (3) negative effects of cannabis; and (4) benefits of recovery while using cannabis.

**Table 1:** Characteristics of Individuals who Resolved an Opioid or Stimulant Addiction and Increased their Cannabis Use

Participant*	Race, Gender, Age	Primary Addiction	Years of Addiction	MOUD	Months addiction resolution	Self-identified psychiatric conditions (*medication from doctor)	Substances used since resolving addiction
Russel	White male, 40	Opioids	20	Sublocade	7–9	Trauma Panic attacks*	Alcohol Cannabis Psychedelics
Kelly	White woman, 45	Methamphetamine	Meth- 1 year; alcohol adult life		5–6	Bipolar disorder Trauma	Alcohol Cannabis
Jimmy	White male, early 40s	Methamphetamine	7		0.5	Trauma, depression, night terrors	Cannabis
Maya	White woman, 24	Methamphetamine	7		9	Insomnia*	Cannabis Methamphetamine (reduced use)
Ian	White male, 32	Opioids/ Crack	17 (including 5 years of abstinence)	Methadone	2	PTSD, ADD	Cannabis
Eric	White male, early 30s	Opioids	8		2	Anxiety, depression, PTSD, anger	Cannabis Methamphetamine (reduced use)
Simon	White male, 27	Opioids	10	Methadone	11	Depression	Cannabis
Jessica	White woman 40	Opioids	12	Methadone	2	Anxiety, panic attacks	Cannabis
Terry	White woman 37	Opioids Cocaine	18	Methadone	12	Bipolar disorder	Benzodiazepines (until Month 10) Cannabis
Kathryn	White woman 20s/ 30s	Opioids	8	Vivitrol	2	Depression*	Cannabis
Omar	Black Somali male, 24	Opioids	2		4	ADHD*	Alcohol Cannabis
Marco	Hispanic male, late 20s	Opioids	7+	Suboxone Gabapentin	5		Alcohol Cannabis
Sam	White male, 25	Opioids, methamphetamine, crack cocaine	3		3		Alcohol Cannabis
Ava	Hispanic woman, early 20s	Opioids	2		3	Bipolar disorder*	Alcohol Cannabis Stimulants (Cocaine/ Ritalin)

*Participant names are pseudonyms

### A better alternative: relatively safe, legally accessible, & socially acceptable

All participants believed cannabis was a safe alternative to other drugs. Maya explained that cannabis, even when illicitly procured, was unlikely to be contaminated, making it safter and more reliable than methamphetamine: “Cannabis is pretty safe like as far as adulteration and you know illicit drug use, or whatever. Like, I know what I’m actually putting into my body when I use it, which is a big deal.” Many participants pointed to the relatively lower risk profile of cannabis as one reason for substitution. Sam had previously used synthetic opioids and research chemicals (i.e., unclassified drugs with unpredictable effects) that he purchased online: “All the other drugs, I was doing had serious consequences, and could absolutely kill you during your use. So, I think it was kind of a relief to do something that was safe and kind of fun.” Sam said he, “couldn’t afford to screw up [his] life anymore,” and was relieved that cannabis offered a safer alternative.

Unlike opioids or stimulants, many participants procured cannabis legally. Jessica purchased cannabis from a medical dispensary: “Weed isn’t like a drug. Not like that. I have my prescription card, my medical marijuana card. I went to a doctor about it.” Using cannabis for medical purposes differentiated it from her previous injection opioid use. Terry lived in a state with recreational cannabis and she purchased it from dispensaries: “And marijuana is legal. You know? So, it’s like I consider myself sober as long as I’m not on any illicit street drugs.” Acquiring cannabis legally informed participants’ conceptualization of cannabis as materially different from their previous substance use.

A few participants attributed their beliefs about cannabis to their family of origin’s beliefs about the substance. Russell stated, “Weed was never presented as like a drug to me. People have always smoked weed. My family smoked…It’s not, it’s not looked at like alcohol or even cigarettes.” Terry and Kelly shared similar stories about their families’ beliefs about cannabis. Familial endorsement differentiated it from illicit street drugs, and even from alcohol, as a safe, non-addictive drug that did not interfere with their recovery.

### Cannabis use is motive driven

#### Replacing other drugs

All participants reported that cannabis helped them avoid using opioids or stimulants. They described this replacement as taking at least three forms: 1) to cope with the cravings for another drug, b) to mimic the effects of another drug, and c) to replace the ritualistic features of other drug use.

Russell explained that cannabis did not prevent cravings, but muted their intensity:

[The cravings are] not completely gone, but they’re tolerable, and I can deal with them… [Using opioids] just doesn’t sound like as good an idea anymore. You know it doesn’t seem like it’s a, it seems like more of a want than a need. You know, like that would be nice if I had some drugs, but I just don’t really feel like going to do that, right now. You know, rather than I *need* to go get some drugs.

Omar had a similar experience: “Yeah, I’ve pretty much had [cravings] daily and then after [I use] the cannabis, the optimistic sense kind of hits me, and it has been like, ‘Oh I don’t actually need [the opioids].’” Cannabis improved his mood enough so that he could reevaluate his desire to use opioids.

When Jimmy experienced cravings for methamphetamine and used cannabis instead, his cravings were entirely relieved: “It’s good for, for curing cravings. I don’t think about, I honestly, after that initial getting stoned, I don’t think about speed. That’s, that’s a big thing. Like I don’t think, ‘God, I really need to hit right now.’” His infrequent cannabis use meant that he experienced its intoxicating effects more acutely, likely helping him pass through the initial cravings. Jessica used cannabis frequently and believed it prevented the onset of opioid cravings:

It pretty much took [the cravings for opioids] away because I would, I would get high [on cannabis], and I would be relaxed. And I get hungry and [am] able to sleep. And as long as I could do all those things, I’m fine.

The physical effects of cannabis mimicked some of the desired effects of opioids (e.g., relaxation, sleepiness), thus reducing her need to use opioids. Like Jessica, Terry described cannabis as a replacement: “The marijuana feeling is mostly a downer feeling, like benzos and heroin and stuff. It’s basically taken place of other drugs. Know what I mean? It’s like a substitution thing.” Cannabis satisfied her desire for the effects of opioids and benzodiazepines well enough so that she could avoid using those substances.

The final way cannabis helped participants avoid using their primary substance was through behavioral rituals. Ava reflected that ritualizing cannabis use served some of the same purposes of her opioid use:

I would say it helped [my recovery]. Because it was something that I could still kind of ritualize, which was like I said a big part of my opiate use. So, it was something that I could still kind of find a ritual in, which is very calming to me.

Replacing opioid-related rituals with cannabis rituals decreased her desire to use them. Simon also ritualized cannabis use. He typically used opioids before and after his evening shift, which he identified as his two “trigger points:”

[Buying cannabis after work] made it easy. Because there was already that concept of like picking up something at night, which I think a lot of drug addiction at some points is…just like the ritual surrounding it…like exchanging money for goods and services. That little monkey part of my brain was like, ‘Alright, cool. We’re satisfied.’

Simon believed that continuing some of the same drug-related behaviors (e.g., procurement after work, using as a reward at the end of the day), helped him avoid using opioids.

#### Regulating affect

All but one participant explained that cannabis use helped regulate their mood. Many described disabling anxiety and attempting to manage the symptoms with cannabis. Jimmy was in the first few weeks of recovery from methamphetamine addiction and described emotional lability, extreme fatigue, and disrupted sleep. He smoked cannabis to soften the moments that were “very prickly, like sharp and hard to deal with”:

I do think the weed helps me at least relax my mind enough to say, ‘You know what yeah, okay, this is something we need to take care of. You’re okay, right now, nothing is crashing down on you because of this.’

Many participants shared this desire - to reduce perseveration and anxiety. Ava was diagnosed with bipolar disorder and did not believe her medication reduced her symptoms to a tolerable level. Cannabis dampened some of the remaining symptoms: “My brain is just always very loud. I usually have a lot of thoughts going on at one time, so [cannabis] kind of just slows everything down, makes everything a little bit more manageable for me.” The motivation to reduce psychiatric symptoms with cannabis could be described as self-medication. For example, Maya explained that her anxiety and social phobias prohibited her from going to the grocery store; but when she used cannabis beforehand, she could complete her tasks with less worry:

It’s easier to be in the moment I guess instead of [wondering]… ‘What do [the staff at the grocery store] think? What am I doing this wrong?’ This that, like all these, like freaking out in every direction about how others are perceiving me.

In this case, Maya was describing using cannabis instead of benzodiazepines to manage anxiety; she did so because cannabis had fewer negative consequences. She acknowledged that some of her anxiety and paranoia were due to her continued methamphetamine use: “I mean, yeah, like literally [this panic has] happened, regardless of whether or not I’m on speed. It usually does if I’m on speed.” She also described similar experiences without using methamphetamine. Participants were not always clear whether symptoms were negative drug-induced side effects or endogenous psychiatric conditions. Regardless, they reported that cannabis use mitigated their anxiety and improved their functioning.

Work was a frequent external stressor that provoked anxiety and led to cannabis use. Eric recently started a new job canvassing and found the work challenging. Smoking after work helped him calm down: “[Cannabis] makes me a little bit less crazy. It makes the anxiety and like racing thoughts like drift away and I’m like - it just helps me like relax after like a long like f-cking stressful day.” Jessica recently quit a telemarketing job due to the stress, however while still employed, she reported using cannabis throughout the day to ease her discomfort: “You’re getting yelled at constantly, getting hung up on… It feels much better when you get to go to the car and smoke a bowl. You know, and then you’re a lot more relaxed and it’s easier to deal with the 200 phone calls.” Omar was frequently responsible for family tasks and stated that completing errands for his mother was a major stressor.

And [my mom] can get angry and be very vocal if I make any mistakes. So sometimes I’ll be nervous to complete the job and make sure I don’t make any mistakes. But if I smoke first then I’ll kind of be more into the flow and end up making less mistakes. So, it’s like yeah, this sense of optimism is - comes from a sense of less anxiety.

Omar believed that by reducing his anxiety, cannabis allowed him to complete tasks effectively with less distraction.

#### Avoiding boredom

Participants consumed cannabis when they wanted distraction from boredom or were completing uninteresting tasks. Russell described this as a long-term strategy to motivate him through monotonous tasks: “[I smoke more] if I’m like doing yard work and sh-t like that, or monotonous like physical labor. There’s nothing like being high and having to like clean the house or do the dishes, like it makes it so much easier.” Participants struggled to manage boredom, whether limited to specific tasks or more generalized boredom. Kathryn attended an intensive outpatient program each morning but had few other obligations; she described her discomfort with managing unstructured time: “Smoking [cannabis] helps with that. It makes it not so hard because, just like my brain is so much more clear than it was before that it’s hard to just do like mundane things. So, sitting around and doing nothing is like hard.” Kathryn stated she was offered a job and believed working would lead to reduced cannabis use.

### Negative effects of cannabis

Though all participants believed cannabis positively impacted their recovery, many also reported negative side effects. Whereas several people reported cannabis minimized their anxiety, increased social anxiety was the most common negative side effect. Eric explained, “It does kind of take me out of it and make it a little harder for me to connect with people, I think. Like puts you on a different plane of understanding and you get a little anxiety accompanying that.” Sam also experienced increased social anxiety: “With cannabis, the bad effects are, for me, mainly my social anxiety becomes worse. I get too caught up in my own thoughts. Like trains of thought will run on when I don’t want them to.” To prevent this, Sam rarely used cannabis in social situations unless he was with close friends.

Some participants said that cannabis increased their focus and helped them accomplish mundane or monotonous tasks, however others explained it decreased their motivation and productivity. Decreased focus and energy helped some pass the time, but others experienced these effects as counterproductive. Kathryn stated, “And when I’m high I just think, like, I’m not really on my A game. I’m not thinking as like clearly…. And I just feel like I don’t get much done.” She indicated that cannabis’ dulling effects positively affected her recovery because it helped her manage her spare time, simultaneously it negatively affected her recovery because it limited her clarity of mind. The complex, and at times conflicting, side effects of cannabis made its effect unpredictable.

Using cannabis to replace primary drugs was a common reason that participants increased their use during recovery. A few people explained, though, that using cannabis in response to an opioid craving increased their desire to feel the effects of opioids. Eva compared it to ineffectively scratching an itch:

That feeling of I have an itch but it’s not really being scratched. Because it, you know, obviously doesn’t have the same effects as, like an opiate or something like that. But it’s like just barely enough to keep you like from wanting to do anything else, but then that can also be frustrating.

In Marco’s case, this strategy led to opioid use: “One of the times I did relapse was because I thought I was going to feel better, I took a hit [of cannabis], right. And what it actually did was intensified my thinking to where I was like, ‘Oh now I need to calm down, right.’” Marco was the only participant who shared that he used opioids in response to cannabis use. While not common in this sample, return to use is one substantial risk of cannabis substitution.

### Benefits of recovery while using cannabis

Despite ongoing challenges related to psychiatric conditions and continued substance use, every participant reported meaningful improvements after resolving their primary addiction and increasing their cannabis use. Many had better, more honest relationships with their families. Ava said that she was able, “to be myself in front of my family and friends, because I’m not hiding anything anymore.” Prior to resolving her addiction, Ava concealed her opioid use. However, her family used cannabis together, and she could join them now without having to hide other substance use. In other cases, participants did not describe using cannabis with their families, but had improved relationships with their families because they were less impaired. Terry was happy to spend more time with her family, “And they want to connect with me more too because I’m not fu-ked up.” Ian echoed a similar experience, “I got to get closer to my mom. And my mother-in-law. So that’s been nice.” Strengthening family intimacy was just one of the benefits of their recovery, even while using cannabis.

Participants also reported relief from the elimination of opioid and stimulant-related consequences. Freeing herself from the powerful hold that opioids had over her, Jessica regained her autonomy:

If I’m going to be dope sick, then I’m not coming. You know what I’m saying? Or I’m going and getting that first. Or, or if I get there, and I have to go get it, I’m going to leave in the middle of family dinner. I’m gonna [sic] go get my drugs… Nothing’s going to stand in the way of me getting, of getting right…the opiates they completely control[ed] my life.

Without urgency to acquire money or drugs, she was more accountable to herself and her family. Kelly shared similar relief that she was rid of the effects of methamphetamine addiction:

[Methamphetamine] just takes over your freakin [sic] life, you forget to eat, you forget to sleep. I’ll be like three days in and not realize I haven’t slept yet, and then you know you start seeing things in the corner of your eyes, because you’re sleep deprived and you’re on this major drug. So yeah, it’s a big difference.

Kelly continued to struggle with her mental health and to moderate her alcohol use. Even so, she was relieved to be rid of methamphetamine-induced deprivation and psychosis: “Everything is kind of the same, except my mind is with me.” The relief from consequences related to opioid and stimulant use was described consistently as impactful to participants’ recovery. Maya was proud of her self-directed change:

I don’t feel any shame whatsoever. I’m actually really proud… I rose to the occasion. Like I made choices, like intentional choices. And followed through on those choices to ensure that I can be responsible and trustworthy.

Maya continued to use methamphetamine, but at a decreased frequency and quantity (i.e., a small amount in the morning), and reaped profound benefits related to improved self-concept and stable employment. Kathryn summarized her growth over the past few months, touching upon many of the themes identified above:

I wake up in the morning and like let my dog out, feed my animals and stuff I could not do before, because I was sick all the time… I read three books which I haven’t read any books and, like the last few years. I made friends, which I didn’t have before. I answer my phone. Less fighting with my husband because I’m not trying to sneak out and go get high. A lot of good things, a lot of little things. I got on depression medication, finally, because I went to the doctor.

For some, cannabis use was directly linked to recovery experiences (e.g., Ava spent time with her family, which involved cannabis use). For the most part, though, cannabis use benefitted participants’ recovery indirectly. They explained that cannabis use helped them reduce or eliminate their primary substance and tolerate experiences without those substances, via replacement, affect regulation, and avoiding boredom; this elimination or reduction facilitated their myriad positive outcomes.

## Discussion

This study identified participants’ motives for, experiences with, and reflections on cannabis use after resolving a primary opioid or stimulant addiction. Participants illustrated cannabis’ host of functional roles. They assessed the risks of cannabis use in comparison to the risks of their previous opioid and methamphetamine use and reasonably concluded that cannabis substitution was substantially less harmful and facilitated progress in their recovery.

### Relative risk

In the absence of a safe drug supply, universal healthcare, and access to safe use supplies, individuals with addictions to opioids and/or stimulants face some of the greatest risks for health and social harms related to drug use, risks that are not attributable to cannabis use [32]. Chronic opioid and methamphetamine use are associated with severe health consequences, including impaired memory and cognition; structural brain changes; increased impulsivity and violent behavior; anxiety, delusions, hallucinations, and psychosis; heart attacks, seizures, liver and kidney damage, and death [33, 34]. Some of these changes are permanent, or persist into a period of abstinence [35]. Additionally, opioid use continues to be a leading cause of drug-related deaths in the U.S., thus, any decrease in opioid use increases survival likelihood [36].

While cannabis substitution may reduce mortality and morbidity related to opioid or stimulant use, cannabis use is not without acute and long-term risk, especially for youth or pregnant people [37, 38]. Acute side-effects include impaired non-verbal learning and memory, attentional control, and motor inhibition, however these side effects generally subside after a period of abstinence [37, 38]. However, the changing drug supply may challenge the validity of these findings, as the average THC concentration has increased annually since 1970 [39]. While research on high potency THC products is nascent, some has found a correlation between high THC and increased likelihood of cannabis use disorder (CUD), increased “dependence,” and increased side effects including memory impairment and paranoia [40, 41].

### Motivational model and cannabis substitution during recovery

The motivational model was an informative framework in examining participants’ cannabis use. Of the motivational model’s extant motives, “to cope” was the most salient motive for cannabis use during recovery. Cannabis use during recovery supported two types of coping: [1] to regulate affect; and [2] to avoid boredom or negative thought patterns. Notably, using cannabis for pleasure or for social purposes was uncommon in this study. Even more than these motives, participants emphasized that cannabis helped them avoid using opioids or methamphetamine. The motivational model does not include a substitution motive and this study suggests that, while similar to the coping motive, substitution is likely a distinct construct. The motivational model’s “to cope” has typically referred to psychological coping with distress of any kind; in contrast, substitution involves physical and psychological dimensions and is exclusively driven by reduced substance use. Additional research may be helpful to determine whether these are different factors.

### Motives for use

#### Substitution

In line with previous research, participants believed that cannabis use protected them from returning to their primary substance or former pattern of use, in part because cannabis helped them manage cravings [32]. In studies on the effect of cannabis use on the return to opioid use by individuals taking medication for opioid use disorder (MOUD), cannabis was associated with decreased likelihood of opioid, alcohol, or cocaine use [42, 43]. In one study, experiencing euphoria or being “high” was associated with decreased likelihood of any opioid use. In the current study, cannabis intoxication did not change participants’ assessment of cannabis’ effectiveness as a deterrent to opioid or methamphetamine use. For example, some participants benefitted from their cannabis use rituals, which were unrelated to its psychoactive effects.

#### Affect regulation

Reducing anxiety was the most reported mood-related motive for cannabis use, suggesting some degree of self-medication [44, 45]. Many participants in this study reported complex psychiatric disorders and previous trauma, which they aimed to treat with cannabis. In many cases participants described high frequency of use to mitigate these symptoms. Further exploration of self-medication with cannabis use is warranted to discern whether it can be exclusively therapeutic, or whether there are always ancillary motives and/or effects. Other participants experienced increased anxiety, especially social anxiety, after using cannabis. Research on cannabis and anxiety reflects these mixed outcomes. A review on this topic found some evidence that cannabis has anxiolytic effects, though many studies had inverse or null results [46]. While cannabis is likely to be inadequate to treat patients’ anxiety without additional mental health intervention, many participants in this study indicated skillful use of cannabis by moderating use according to its effects (e.g., only using cannabis with close friends to avoid increased social anxiety).

#### Boredom

Boredom, the aversive state due to a monotonous environment and difficulty remaining engaged with the environment, is a natural part of early recovery. Boredom and even the anticipation of boredom are known barriers to entering or staying in recovery [47–49]. In the general population, people use cannabis to mitigate boredom [50, 51] yet there is little research on the relationship between boredom and recovery and how cannabis use interacts with these states. In the present study, participants described the connection between cannabis use and boredom in three ways: first, cannabis helped them accomplish tasks in which they had little interest; second, it ameliorated negative emotional experiences prompted by boredom; and third, it helped them tolerate boredom produced by unstructured time, in many cases due to unemployment.

Unemployment is common in early recovery and increased participation in the workforce often occurs over time in recovery [52, 53]. For those in early recovery, engagement with community, work, or hobbies increases recovery capital and diminishes boredom [54]. Employment interventions in abstinence-based treatment settings have been associated with positive substance use outcomes [55]. However, many individuals like those in this study do not have clear pathways to access employment, nor are they embedded in peer support communities, which often help people re-enter the workforce. This barrier to services and recovery capital points toward an important area for future intervention development.

### Implications for conceptions of recovery

Research increasingly acknowledges that recovery includes both abstinent and non-abstinent paths, paths which may vary by addiction and psychiatric severity, complexity, and chronicity [2, 20, 56]. Yet, there are few treatment or mutual aid settings where non-abstinent individuals can access the recovery resources available to their abstinence-seeking peers, as such settings view cannabis use as incompatible with recovery.

Due to potential medical use, cannabis use may or may not violate the principle of abstinence from non-prescribed psychoactive substances. Taking cannabis to treat a condition (e.g., chronic pain, posttraumatic stress disorder, chemotherapy-induced nausea) may be categorically closer to taking a prescription stimulant for Attention-deficit/hyperactivity disorder (ADHD) than to using cannabis recreationally. However, unlike medication for ADHD, there are no dosing guidelines or maximum dosing thresholds [57]. Without parameters for use, it remains challenging to classify cannabis use as strictly medical. Further study on the role of medical cannabis use in recovery is warranted to understand whether its use is compatible with the construct of abstinence.

Many participants reported previous treatment or 12-step participation, noting that these settings viewed their goals as incompatible with the settings’ conceptualization of successful recovery [56]. Upholding the belief that abstinence is the foundation of recovery, as many treatment and mutual aid settings do, discounts the substantial growth and improvement of people who, despite non-abstinence, recover from their addictions [2]. Equating abstinence with recovery reinforces stigmatizing conceptualizations of this population by differentiating between those who have and have not changed their substance consumption “enough,” or those who do and do not count as “recovered” [58, 59]. Without embracing a more inclusive recovery paradigm, individuals like the participants in this study will continue to be perceived as “less well” and will continue to have fewer options for medical and mental health support compared to their abstinence-seeking peers [60].

### Study limitations

Findings should be interpreted in the context of these limitations. First, this preliminary study on cannabis substitution was conducted with a small sample, which may mean that conceptual categories integral to non-abstinence recovery with substitution were missed [25]. Future qualitative studies on this topic should aim for larger samples and could consider the addition of quantitative measures. Recruiting via social media sites was effective in accessing a hard-to-reach population, but the resultant sample was limited to individuals who were aware of and engaged with these sites. The sample was predominantly White, which possibly reflects the demographics of individuals using Reddit, the recruitment site for most participants. Clinical settings, including primary care and addiction specific clinics, may be useful settings for recruitment in future studies. The interview did not explicitly ask about mental health history and likely missed some participants’ diagnoses and psychiatric medication. Although participants described their substance use history, they did not complete a clinical intake and thus their drug-use severity is unknown. In future work, researchers should collect precise data about mental health and addiction history to improve the understanding of who substitutes with cannabis and under what circumstances. Finally, this study was a single point in time and longitudinal studies are critical to understanding whether substitution and its effects change over time.

## Conclusion

This study increases our insight about cannabis substitution in early recovery, documenting its potential roles during this period. At this time, cannabis’ relatively lower- risk profile makes it an effective harm reduction strategy for those in early recovery from an opioid or stimulant addiction [7, 32]. Future studies are needed to assess the degree to which this substitution strategy is sustainable over time, as well as the later risks for primary addiction recurrence or development of a cannabis use disorder. Taking a harm reduction approach to drug use and addiction recovery has the potential to positively transform this population’s recovery experiences and willingness to seek support [61, 62].

## Data Availability

No datasets were generated or analysed during the current study.
